# Supplementary material for: The Evolution and Characterization of the RNA Interference Pathways in Lophotrochozoa
Source: Genome Biol Evol. 2024 May 7;16(5):evae098. doi: 10.1093/gbe/evae098 (PMC11114477; doi:10.1093/gbe/evae098)
Supplement: evae098_Supplementary_Data [file evae098_supplementary_data.zip › Supplementary_materials.zip!/selected_miRNAs/NoGe_selected_miRNAs/known_miRNA_families_pdf_files/NMRB01002226.1_Notospermus_geniculatus_strain_Ushimado_scaffold2226,_whole_genome_shotgun_sequence_36874.pdf]

Provisional ID	:	NMRB01002226.1_Notospermus_geniculatus_strain_Ushimado_scaffold2226,_whole_genome_shotgun_sequence_36874
Score total	:	2107
Score for star read(s)	:	3.9
Score for read counts	:	2098.6
Score for mfe	:	1.5
Score for randfold	:	0
Score for cons. seed	:	3
Total read count	:	4128
Mature read count	:	4099
Loop read count	:	0
Star read count	:	29

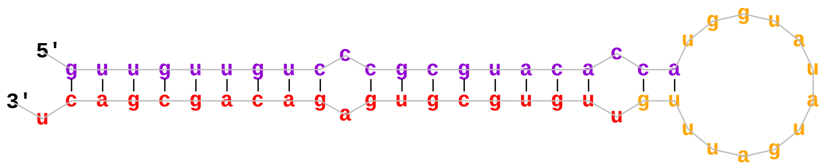

Genome coordinates: NMRB01002226.1 101828-101883

pre-miRNA sequence:
GUUGUUGUCCCGCGUACACCAGUUGUUGUCCCGCGUACACCAUGGUAUAUGAUUUGUUGUGCGUGAGACAGCGACU

Mature sequence: UUGUGCGUGAGACAGCGACU

Aligned mature sequence of the *N. geniculatus* pre-miRNA with the top 5 hits from the BLAST search against the pre-miRNA mirGeneDB set:

```
                                   *******************
    Nge_Mir-210-P16                uugugcgugagacagcgacu-
    Obi-Mir-210-P16_3p             uugugcgugagacagcgaccg
    Ovu-Mir-210-P16_3p             uugugcgugagacagcgaccg
    Esc-Mir-210-P16_3p             uugugcgugagacagcgaccg
```
